# Mechanomemory after short episodes of intermittent stresses induces YAP translocation via increasing F-actin

**DOI:** 10.1063/5.0253046

**Published:** 2025-04-18

**Authors:** Fazlur Rashid, Elvis Njoki, Sadia Amin Kabbo, Ning Wang

**Affiliations:** 1 The Institute for Mechanobiology, Northeastern University, Boston, Massachusetts 02115, USA; 2 Department of Bioengineering, College of Engineering, Northeastern University, Boston, Massachusetts 02115, USA; 3 Department of Mechanical Science and Engineering, The Grainger College of Engineering, University of Illinois at Urbana-Champaign, Urbana, Illinois 61801, USA

## Abstract

How forces and mechanics influence and regulate living cells remains elusive. Mechanomemory, the response to a mechanical perturbation that persists after the perturbation is removed, is believed to be a key to understanding the impact of forces and mechanics on cell functions. Recently, our lab has demonstrated the presence of mechanomemory that lasts for ∼30 min after applying external stress via integrins. Herein, we test the hypothesis that applications of short intermittent episodes of stress exert long-term effects on mechanomemory via the process of mechanotransduction. An Arginine-Glycine-Aspartic acid (RGD)-peptides-coated 4-*μ*m magnetic bead was bound to the integrin receptors to apply stresses to the surface of a Chinese Hamster Ovary cell. At the same stress magnitude and frequency (15 Pa at 0.3 Hz), multiple cycles of externally applied intermittent 2 or 10 min stresses with 15 min intervals, 10 min stresses with 10 min intervals, or a 30 min stress plus a 30 min load-free interval increased nuclear translocation of YAP (Yes-Associated Protein) and *Ctgf* gene expression, like that by a 60 min continuous stress, but a 30 min continuous stress did not. Short durations of intermittent stresses increased F-actin in the cytoplasm, which coincided with the elevated YAP translocation. Inhibiting F-actin or actomyosin but not microtubules blocked stress-induced YAP translocation to the nucleus. Cells on soft substrates translocate more YAP than on stiff substrates after external load release. These results highlight the impact of multiple intermittent stresses-induced cytoplasmic mechanomemory on cell biological functions via YAP translocation.

## I. INTRODUCTION

Mechanomemory is a biological response to mechanical perturbation long after the cessation of the perturbation. Published reports demonstrate the existence of mechanomemory or mechanoplasticity of the cells after they switch from stiff substrates to soft substrates or vice versa following being on the original substrates for days to weeks.^1–7^ It is reported that the fate of human mesenchymal stem cells (hMSCs) toward osteogenic differentiation depends on mechanomemory of the cells on substrate stiffness, which, in turn, is dependent on the translocation of YAP (Yes-associated protein) and TAZ (transcriptional coactivator with PDZ-binding domain) from the cytoplasm to the nucleus to regulate gene expression.^3^ Recent reports demonstrate the effects of stiffening or softening on mechanomemory of skeletal muscle stem cells,^8^ human mesenchymal stem cells,^9^ and pulmonary fibroblasts.^10^ Different engineering approaches for testing and examining cellular mechanomemory have been summarized in two recent reviews.^11,12^ We have reported that Nupr1 (nuclear protein 1), a YAP-nuclear-translocation-dependent suppressor of growth of highly malignant and tumorigenic tumor-repopulating cells (TRCs), is downregulated in the cells that are seeded for 1 day to 5 days within three-dimensional (3D) soft matrices to promote TRC growth,^13^ independent of self-renewal gene *Sox2*. In addition, we have shown that applied stresses via integrins for 2 min do not induce YAP translocation into the nucleus,^14^ and it takes 60 min continuous mechanical stretching of the substrate to induce YAP translocation to activate its downstream target *Ctgf* (connective tissue growth factor) transcription in the nucleus.^15^ It is known that intermittent forces from muscle contractions occur during exercise. It is reported that intermittent compressive forces applied for several hours per day induce RANKL in human periodontal ligament cells with less cell damage than continuous compressive forces^16^ and that intermittent force-induced YAP promotes osteogenesis but inhibits adipogenesis in these cells.^17^ Recently, we have revealed that 2 or 10 min magnetic bead stresses on the cell surface via integrins^18^ or stresses from an anti-H2B antibody coated microinjected magnetic nanoparticle directly applied to the chromatin^19^ lead to nuclear mechanomemory for ∼30 min after cessation of the applied stress.^18,19^ Because short durations of applied stresses on the cell surface via integrins do not affect YAP translocation, we wondered if intermittent short episodes of applied stresses could have a cumulative impact on YAP translocation due to mechanomemory in the cytoplasm. Herein, we tested the hypothesis that applications of short intermittent episodes of stress exert long-term effects on mechanotransduction via cytoplasmic mechanomemory.

We found that multiple cycles of 2 min stresses with load-free intervals of 15 min or 10 min stresses with load-free intervals of 10 or 15 min for a total duration of ∼60 min or a 30 min stress plus a 30 min pause increased nuclear translocation of YAP/TAZ, like that by a 60 min continuous stress, but a 30 min continuous stress did not. Intermittent short durations of stresses increased F-actin in the cytoplasm, which coincided with the elevated YAP/TAZ translocation. Inhibiting F-actin or non-muscle myosin II but not microtubules blocked YAP translocation to the nucleus. Cells on soft substrates translocate more YAP than on stiff substrates after external load release. These results highlight the importance of cytoplasmic mechanomemory in long-term biological functions of the cells and have implications in mechanomedicine.

## II. RESULTS

### A. Short intermittent stress episodes induce YAP translocation

**FIG. 1.**
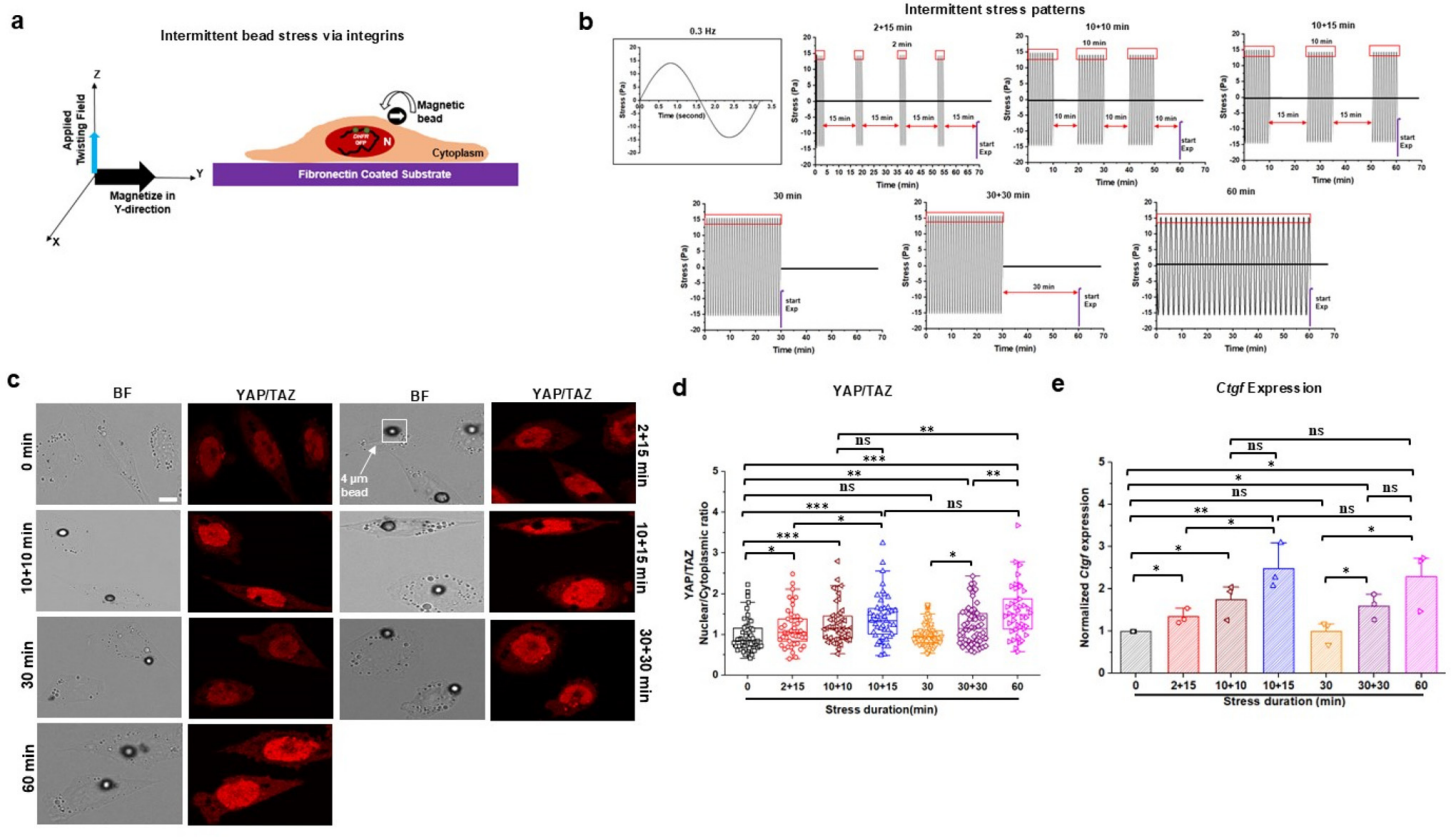
Multiple short intermittent stresses induce YAP/TAZ nuclear translocation via cytoplasmic mechanomemory. (a) Schematic of a 4-*μ*m Arginine-Glycine-Aspartic acid (RGD)-coated ferromagnetic bead binds to the integrin receptors of Chinese Hamster Ovary (CHO) cell to apply stress by magnetizing in Y-direction and applied twisting field (all stresses were applied with 15 Pa at 0.3 Hz) in Z-direction. (b) Multiple sinusoidal stresses of 2 or 10 min with 15 min or 10 min intervals, continuous sinusoidal stresses of 30 or 60 min, and a 30 min interval after 30 min continuous stress to quantify YAP/TAZ nuclear translocation and mechanomemory. (c) Representative brightfield (BF) and YAP/TAZ immunofluorescence (IF; red) images (Anti-YAP/TAZ primary antibody and Anti-Rabbit IgG H&L (Alexa Fluor^®^ 555) secondary antibody) of CHO cells at 0 min (no stress; control), multiple 2 min (2 + 15 min) or 10 min with 10 min (10 + 10 min) or 15 min (10 + 15 min) interval, continuous 30 min or 60 min, and 30 min interval after 30 min continuous (30 + 30 min) stress. The black dot was an RGD-coated ferromagnetic bead. Scale bar, 10 *μ*m. (d) Normalized YAP/TAZ nuclear to cytoplasmic (nuclear/cytoplasmic) intensity ratio for 0, 2 + 15, 10 + 10, 10 + 15, 30, 30 + 30, or 60 min applied stress. Data are shown as boxplot with values of minimum, 5% percentile, 25% percentile, median, 75% percentile, maximum, and mean. n = 55 cells for 0 min, n = 45 cells for 2 + 15 min, n = 48 cells for 10 + 10 min, n = 45 cells for 10 + 15 min, n = 61 cells for 30 min, n = 56 cells for 30 + 30 min, and n = 42 cells for 60 min stress condition from three independent experiments; *P* = 0.019 between 0 and 2 + 15 min; *P* = 2 × 10^−4^ between 0 and 10 + 10 min; *P* = 1.74 × 10^−5^ between 0 and 10 + 15 min; *P* = 0.022 between 2 + 15 and 10 + 15 min; *P* = 2.30 × 10^−7^ between 0 and 60 min; *P* = 0.0013 between 0 and 30 + 30 min; *P* = 0.018 between 30 and 30 + 30 min; *P* = 0.0034 between 30 + 30 and 60 min; *P* = 0.008 between 10 + 10 and 60 min. (e) qPCR analysis of *Ctgf expression for* 0, 2 + 15, 10 + 10, 10 + 15, 30, 30 + 30, or 60 min applied stress. Data are shown as bar plot with mean ± s.e.m. values from three independent biological experiments. *P* = 0.014 between 0 and 2 + 15 min; *P* = 0.019 between 0 and 10 + 10 min; *P* = 0.005 between 0 and 10 + 15 min; *P* = 0.01 between 0 and 60 min; *P* = 0.013 between 0 and 30 + 30 min; *P* = 0.027 between 2 + 15 and 10 + 15 min; *P* = 0.033 between 30 and 30 + 30 min; *P* = 0.021 between 30 and 60 min. ^*^P < 0.05, ^**^P < 0.01, ^***^P < 0.001; ns, not significantly different. All *P* values were quantified using one-way ANOVA with Tukey's test and Mann–Whitney test.

To determine if application of short durations of applied stresses can lead to relatively longer biological effects in the cell, we attached RGD (Arg-Gly-Asp)-peptide-coated magnetic beads (4-*μ*m in diameter) to the surfaces of CHO (Chinese Hamster Ovary) cells that were plated overnight (12 h) on a Fibronectin-coated rigid dish [Fig. 1(a)]. To avoid force interactions between two or more magnetic beads, a single bead on an individual cell was chosen for all experiments. After ∼30 min of bead attachment on the cell surface, the loosely bound beads were washed out of the dish with fresh medium. The magnetic bead was magnetized along the Y-direction with a strong pulse (1000 Gauss for 10 ms) of the magnetic field and then twisted along the Y-Z plane via a weak sinusoidal magnetic field (50 Gauss at 0.3 Hz) that did not re-magnetize the bead but generated a rotational movement of the bead because the bead was ferromagnetic and maintained its magnetic moment after magnetization [Fig. 1(a)]. Then, 6 different patterns of stress durations were applied: (1) four episodes of intermittent 2 min stresses with 4 intervals of 15 min of load-free period for a total duration of 68 min (denoted as 2 + 15), (2) three episodes of intermittent 10 min stresses with 3 intervals of 10 min of load-free period for a total duration of 60 min (denoted as 10 + 10), (3) three episodes of intermittent 10 min stresses with 2 intervals of 15 min of load-free period for a total duration of 60 min (denoted as 10 + 15), (4) one 30 min continuous stress, (5) one 30 min stress with a 30 min load-free period for a total duration of 60 min (denoted as 30 + 30), or (6) one 60 min continuous stress as positive control because it is known that 60 min stress or stretch is long enough to induce YAP translocation^15^ [Fig. 1(b)]. The choice of 10 min, 15 min, or 30 min load-free intervals was based on our published results that mechanomemory lasts for ∼ 30 min.^18,19^ Because the stress amplitude and the frequency were the same (15 Pa at 0.3 Hz) for all patterns, these stress duration patterns could be used to examine the effects of mechanomemory and total mechanical energy per volume (i.e., stress) inputs on the cellular mechanotransduction process of YAP/TAZ translocation. Compared to the no stress control (0 min stress), the ratio of nucleus to cytoplasm of YAP/TAZ was increased for 2 + 15, 10 + 10, and 10 + 15 conditions but not for the 30 min stress condition [Figs. 1(c) and 1(d)]. However, YAP/TAZ translocation was increased by the 30 + 30 min stress compared to zero-stress control. As expected, 60 min continuous stress led to elevated YAP translocation. The total duration of four episodes of intermittent 2 min stresses was only 8 min, much shorter than the 30 min continuous stress. Additional results from subcellular fractionation showed that intermittent stresses of 2 + 15, 10 + 10, 10 + 15, and 30 + 30, but not 30 min alone, induced YAP nuclear translocation [supplementary material, Figs. S1(a) and S1(b)], which in turn activates its downstream *Ctgf* gene expression [Fig. 1(e)] and protein synthesis [supplementary material, Figs. S1(c) and S1(d)]. These results suggest that it took time for the YAP/TAZ to be activated and translocated by applied stress, and mechanomemory in the cytoplasm might have played a key role in the translocation.

### B. Elevated F-actin in response to stress contributes to YAP translocation

**FIG. 2.**
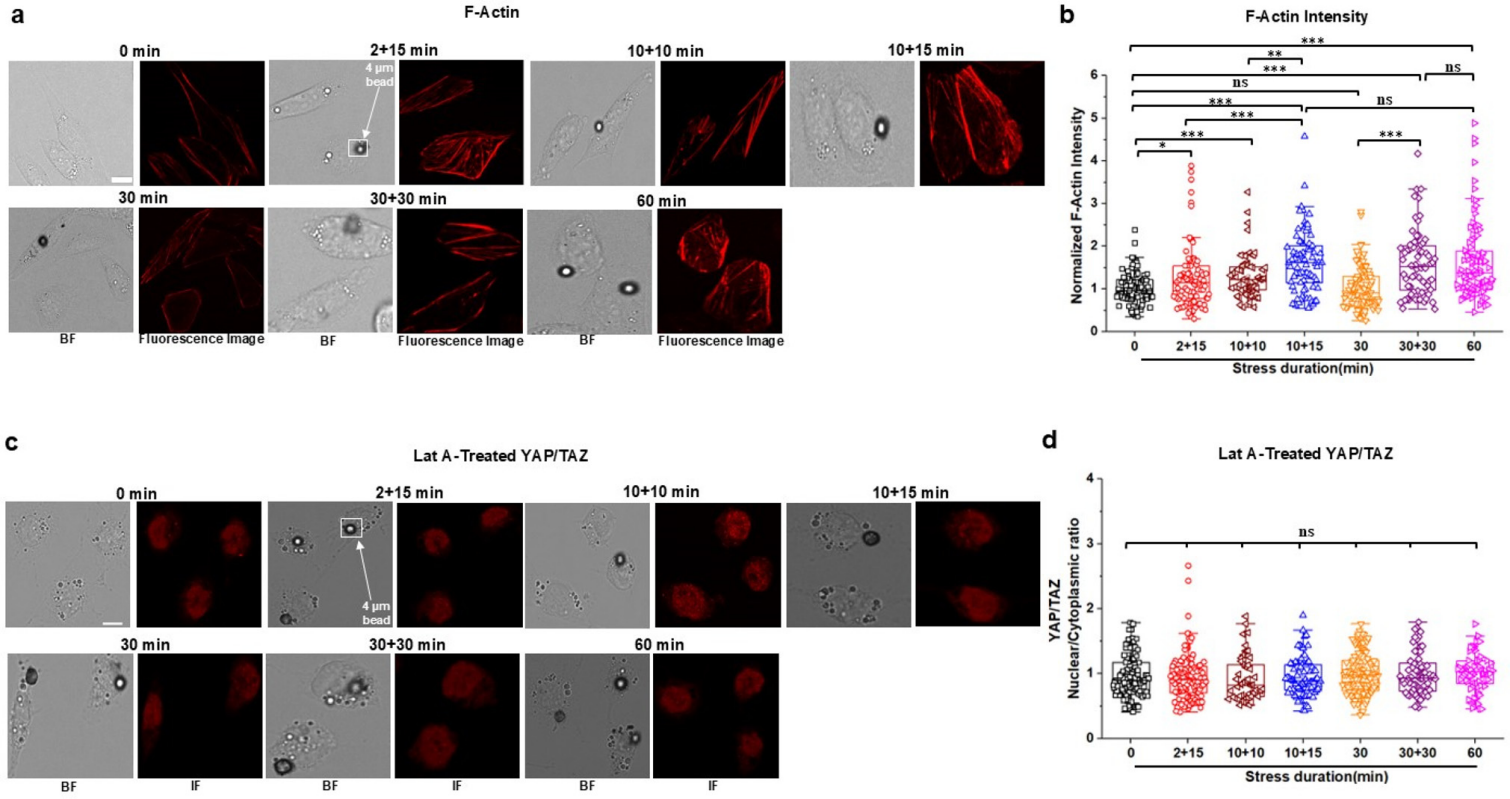
Multiple short intermittent stresses increase F-actin and disrupting F-actin abolishes YAP/TAZ translocation. (a) Representative brightfield (BF) and F-actin stained (SiR-actin) fluorescence images (red) of live CHO cells at 0 min (no stress; control), multiple 2 min (2 + 15 min) or 10 min with 10 min (10 + 10 min) or 15 min (10 + 15 min) interval, continuous 30 min or 60, and 30 min interval after 30 min continuous stress (30 + 30 min); all stresses were 15 Pa at 0.3 Hz. Circular black dots are RGD-coated ferromagnetic beads. Scale bar, 10 *μ*m. (b) Normalized F-actin fluorescence intensity for 0, 2 + 15, 10 + 10, 10 + 15, 30, 30 + 30, or 60 min applied stress of 15 Pa at 0.3 Hz. Data are shown as boxplot with values of minimum, 5% percentile, 25% percentile, median, 75% percentile, maximum, and mean. n = 105 cells for 0 min, n = 87 cells for 2 + 15 min, n = 57 cells for 10 + 10 min, n = 77 cells for 10 + 15 min, n = 115 cells for 30 min, n = 64 cells for 30 + 30 min, and n = 99 cells for 60 min stress condition from three independent experiments; *P* = 0.032 between 0 and 2 + 15 min; *P* = 0.0001 between 0 and 10 + 10 min; *P* = 2.46 × 10^−8^ between 0 and 10 + 15 min; *P* = 5.34 × 10^−5^ between 2 + 15 and 10 + 15 min; *P* = 1.50 × 10^−8^ between 0 and 60 min; *P* = 0.014 between 2 + 15 and 30 min; *P* = 0.0011 between 2 + 15 and 60 min; *P* = 4.50 × 10^−8^ between 10 + 15 and 30 min; *P* = 3.26 × 10^−8^ between 30 and 60 min; *P* = 8.22 × 10^−8^ between 0 and 30 + 30 min; *P* = 0.0011 between 2 + 15 and 30 + 30 min; *P* = 2.01 × 10^−8^ between 30 and 30 + 30 min; *P* = 0.0032 between 10 + 10 and 10 + 15 min. (c) Representative brightfield (BF) and YAP/TAZ immunofluorescence (IF; red) images (used YAP/TAZ antibody and Anti-Rabbit IgG H&L (Alexa Fluor® 555) antibody) of CHO cells treated with 1 *μ*M Latrunculin A (Lat A) for 30 min at 0 min (no stress; control), multiple 2 min (2 + 15 min) or 10 min with 10 min (10 + 10 min) or 15 min (10 + 15 min) interval, continuous 30 min or 60, and 30 min interval after 30 min continuous (30 + 30 min) stress of 15 Pa at 0.3 Hz. Circular black dots are RGD-coated ferromagnetic bead. Scale bar, 10 *μ*m. (d) Normalized YAP/TAZ nuclear to cytoplasmic (nuclear/cytoplasmic) intensity ratio in 1 *μ*M Latrunculin A (Lat A)-treated CHO cells for 0, 2 + 15, 10 + 10, 10 + 15, 30, 30 + 30, or 60 min applied stress. Data are shown as boxplot with values of minimum, 5% percentile, 25% percentile, median, 75% percentile, maximum, and mean. n = 102 cells for 0 min, n = 111 cells for 2 + 15 min, n = 46 cells for 10 + 10 min, n = 76 cells for 10 + 15 min, n = 115 cells for 30 min, n = 63 cells for 30 + 30 min, and n = 65 cells for 60 min stress condition from three independent experiments. ^*^P < 0.05, ^**^P < 0.01, ^***^P < 0.001; ns, not significantly different. All *P* values were quantified using one-way ANOVA with Tukey's test and Mann–Whitney test.

**FIG. 3.**
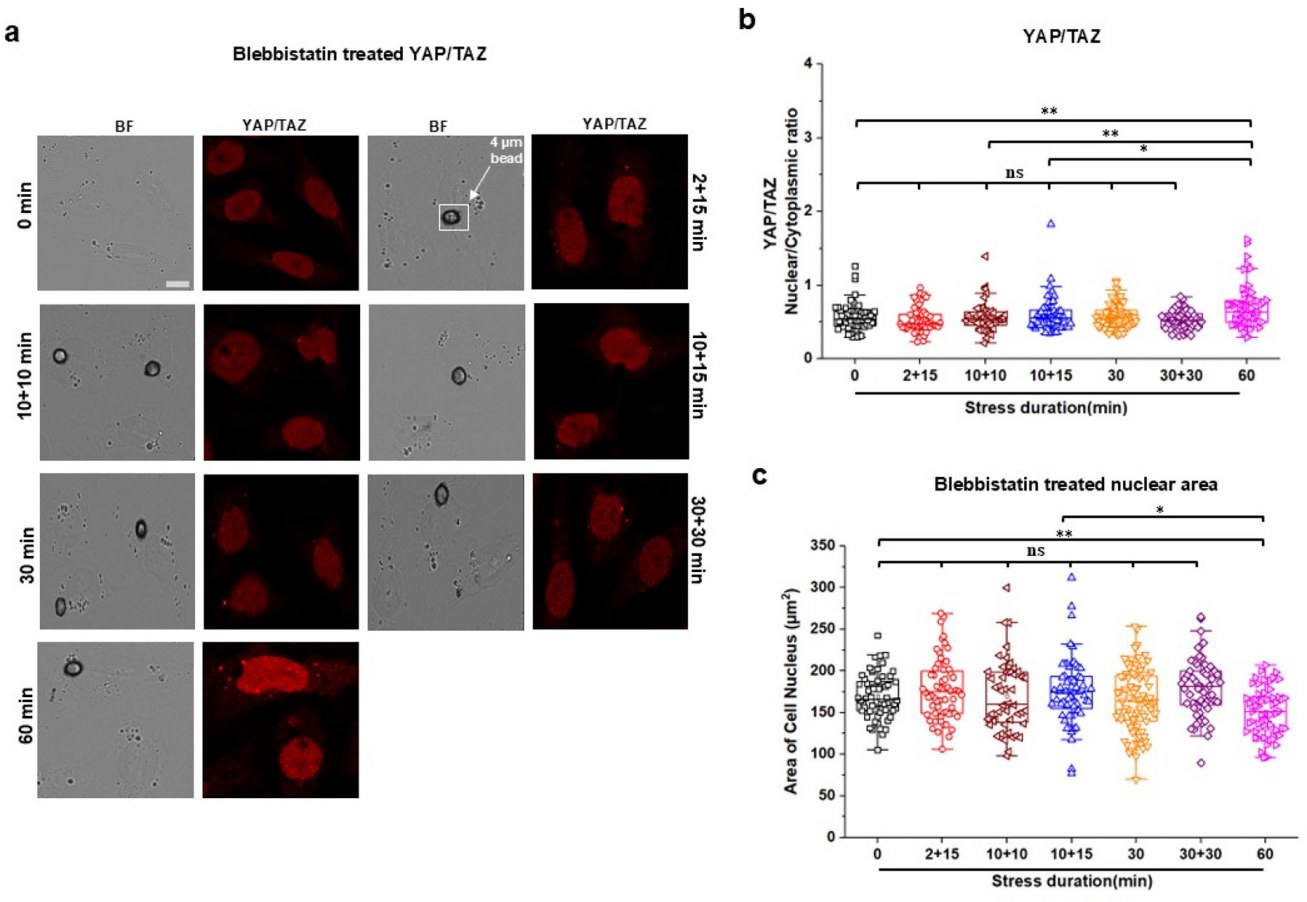
Inhibiting non-muscle myosin II block mechanomemory-mediated YAP/TAZ nuclear translocation. (a) Representative brightfield (BF) and YAP/TAZ immunofluorescence (IF; red) images (used YAP/TAZ antibody and Anti-Rabbit IgG H&L (Alexa Fluor® 555) antibody) of CHO cells treated with 20 *μ*M Blebbistatin for 30 min at 0 min (no stress; control), multiple 2 min (2 + 15 min) or 10 min with 10 min (10 + 10 min) or 15 min (10 + 15 min) interval, continuous 30 min or 60, and 30 min interval after 30 min continuous (30 + 30 min) stress of 15 Pa at 0.3 Hz. Circular black dots are RGD-coated ferromagnetic beads. Scale bar, 10 *μ*m. (**b)** Normalized YAP/TAZ nuclear to cytoplasmic (Nuclear/Cytoplasmic) intensity ratio of 20 *μ*M Blebbistatin-treated CHO cells at 0, 2 + 15, 10 + 10, 10 + 15, 30, 30 + 30, or 60 min applied stress of 15 Pa at 0.3 Hz. Data are shown as boxplot with values of minimum, 5% percentile, 25% percentile, median, 75% percentile, maximum, and mean. n = 60 cells for 0 min, n = 60 cells for 2 + 15 min, n = 48 cells for 10 + 10 min, n = 60 cells for 10 + 15 min, n = 85 cells for 30 min, n = 57 cells for 30 + 30 min, and n = 59 cells for 60 min stress condition from three independent experiments; *P* = 0.004 between 0 and 60 min; *P* = 1.59 × 10^−4^ between 2 + 15 and 60 min; *P* = 0.009 between 10 + 10 and 60 min*; P* = 0.023 between 10 + 15 and 60 min; *P* = 0.008 between 30 and 60 min; *P* = 1.19 × 10^−4^ between 30 + 30 and 60 min. (c) Quantified cell nuclear area of 20 *μ*M Blebbistatin-treated CHO cells at 0, 2 + 15, 10 + 10, 10 + 15, 30, 30 + 30, or 60 min applied stress of 15 Pa at 0.3 Hz. Data are shown as boxplot with values of minimum, 5% percentile, 25% percentile, median, 75% percentile, maximum, and mean. n = 60 cells for 0 min, n = 60 cells for 2 + 15 min, n = 48 cells for 10 + 10 min, n = 60 cells for 10 + 15 min, n = 85 cells for 30 min, n = 57 cells for 30 + 30 min, and n = 59 cells for 60 min stress condition from three independent experiments; *P* = 0.0014 between 0 and 60 min; *P* = 0.025 between 10 + 15 and 60 min.^*^P < 0.05, ^**^P < 0.01; ns, not significantly different. All *P* values were quantified using one-way ANOVA with Tukey's test and Mann–Whitney test.

**FIG. 4.**
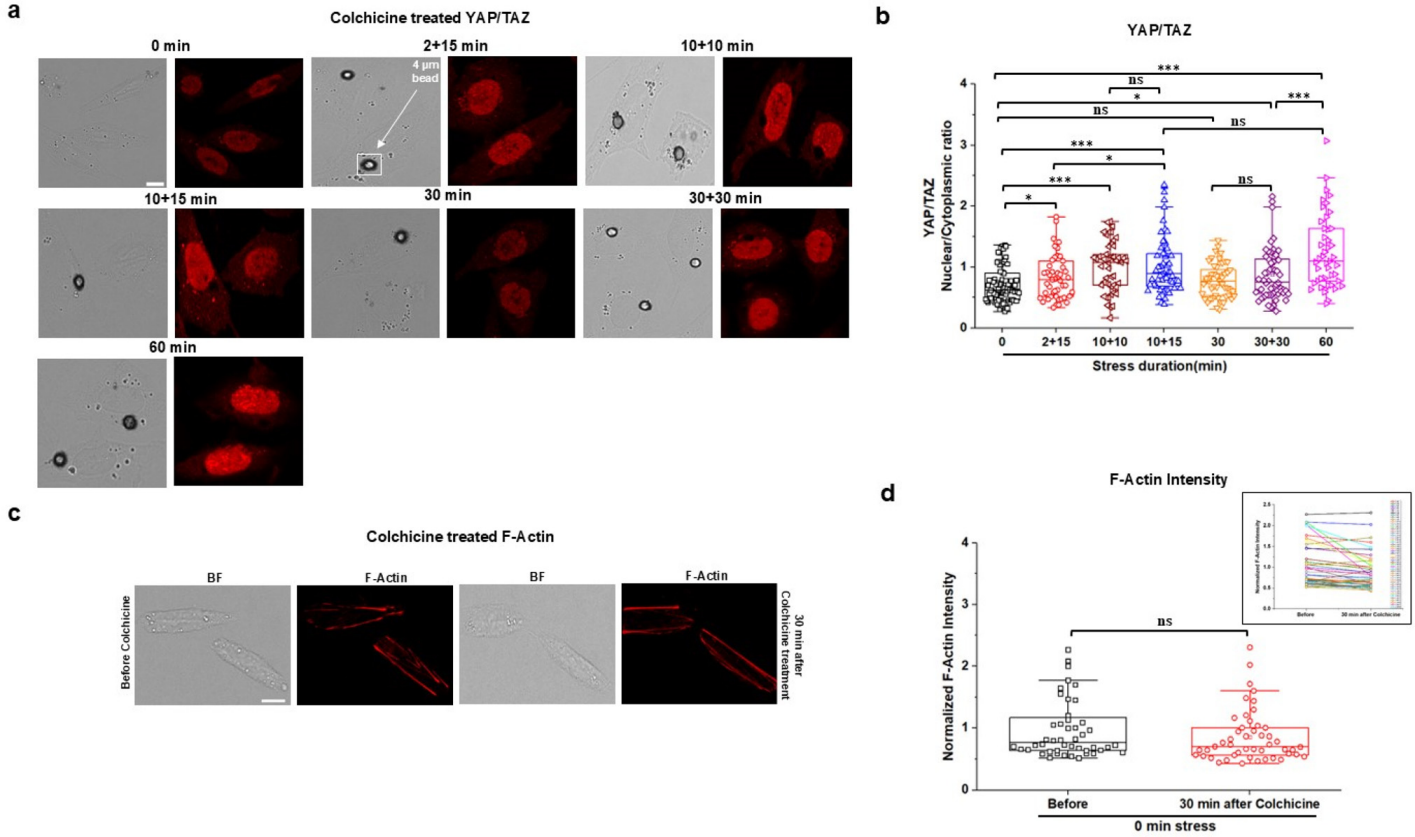
Inhibiting microtubule does not block YAP/TAZ nuclear translocation by disrupting F-actin. (a) Representative brightfield (BF) and YAP/TAZ immunofluorescence (IF; red) images (used YAP/TAZ antibody and Anti-Rabbit IgG H&L (Alexa Fluor^®^ 555) antibody) of CHO cells treated with 10 *μ*M Colchicine for 30 min at 0 min (no stress; control), multiple 2 min (2 + 15 min) or 10 min with 10 min (10 + 10 min) or 15 min (10 + 15 min) interval, continuous 30 min or 60, and 30 min interval after 30 min continuous (30 + 30 min) stress of 15 Pa at 0.3 Hz. Circular black dots are RGD-coated ferromagnetic beads. Scale bar, 10 *μ*m. (b) Normalized YAP/TAZ nuclear to cytoplasmic (nuclear/cytoplasmic) intensity ratio after 10 *μ*M Colchicine-treated CHO cells exposed to 0, 2 + 15, 10 + 10, 10 + 15, 30, 30 + 30, or 60 min applied stress of 15 Pa at 0.3 Hz. Data are shown as boxplot with values of minimum, 5% percentile, 25% percentile, median, 75% percentile, maximum, and mean. n = 79 cells for 0 min, n = 47 cells for 2 + 15 min, n = 49 cells for 10 + 10 min, n = 57 cells for 10 + 15 min, n = 49 cells for 30 min, n = 54 cells for 30 + 30 min, and n = 45 cells for 60 min stress condition from three independent experiments; *P* = 0.042 between 0 and 2 + 15 min; *P* = 2.77 × 10^−5^ between 0 and 10 + 10 min; *P* = 6.43 × 10^−6^ between 0 and 10 + 15 min; *P* = 0.033 between 2 + 15 and 10 + 15 min; *P* = 2.46 × 10^−8^ between 0 and 60 min; *P* = 0.03 between 0 and 30 + 30 min; *P* = 5.53 × 10^−4^ between 30 + 30 and 60 min. (c) Representative brightfield (BF) and F-actin stained (SiR-actin) fluorescence images (red) of live CHO cells at 0 min (no stress; control) before (left two images) and 30 min after (right two images) 10 *μ*M Colchicine treatment. Cells were tracked before and 30 min after 10 *μ*M Colchicine treatment. (d) Normalized F-actin fluorescence intensity of live CHO cells at 0 min (no stress; control) before and 30 min after 10 *μ*M Colchicine treatment. Data are shown as boxplot with values of minimum, 5% percentile, 25% percentile, median, 75% percentile, maximum, and mean. n = 48 cells for before and 30 min after 10 *μ*M Colchicine treatment from three independent experiments. Inset shows F-actin intensity of each individual cell before and 30 min after 10 *μ*M Colchicine treatment. ^*^P < 0.05, ^**^P < 0.01, ^***^P < 0.001; ns, not significantly different. All *P* values were quantified using one-way ANOVA with Tukey's test and Mann–Whitney test.

To determine what contributed to YAP/TAZ translocation in response to short episodes of intermittent stresses, we examined live cell fluorescence intensity of filamentous actin (F-actin) under various patterns of stresses. The F-actin was chosen because it is known as the primary stress-bearing element in the cytoskeleton in response to stresses applied via integrins.^20,21^ Using the same six different patterns of stress durations as those for the YAP/TAZ translocation measurements, we found that short episodes of 2 + 15, 10 + 10, 10 + 15, or 30 + 30 stresses elevated F-actin intensity when compared with the zero-stress control, similar to that by the 60 min continuous stresses [Figs. 2(a) and 2(b)]. However, 30 min continuous stress did not induce changes in F-actin intensity [Figs. 2(a) and 2(b)]. These stress duration results are in accord with the published report that 30 min to 60 min continuous stresses elevate F-actin in airway smooth muscle cells.^22^ These temporal data of intermittent stresses on F-actin mirrored the effects of the short episodes of intermittent stresses on YAP/TAZ translocation. To further examine if the elevated F-actin intensity was the reason for YAP/TAZ translocation, we pretreated the cells with latrunculin A (1 *μ*M for 30 min), a specific inhibitor of F-actin (supplementary material, Fig. S2). Disrupting F-actin completely abolished external stress-induced YAP/TAZ translocation after all short intermittent episodes of stresses as well as the 60 min continuous stress [Figs. 2(c) and 2(d)]. In addition, pretreating the cells with Blebbistatin (20 *μ*M for 30 min), a specific inhibitor of non-muscle myosin II ATPase, which disrupted F-actin [supplementary material, Fig. S3(a)], abolished myosin light chain phosphorylation [supplementary material, Figs. S3(b) and S3(c)], and inhibited traction generation [supplementary material, Figs. S3(d) and S3(e)], resulted in abolishing YAP/TAZ translocation induced by the short episodes of intermittent stresses [Figs. 3(a) and 3(b)]. These results are consistent with the published report that translocation of YAP/TAZ to the nucleus is enhanced by increasing F-actin and actomyosin contractility and is decreased by disrupting F-actin in human pluripotent stem cells with cytochalasin D.^23^ However, after Blebbistatin treatment to inhibit actomyosin contractility, in response to the 60 min continuous stress, there was still a moderate but significant elevation in YAP/TAZ translocation to the nucleus of the cells [Figs. 3(a) and 3(b)]. To examine the potential role of nuclear deformation in YAP translocation, we quantified nuclear areas before and after Blebbistatin treatment and after the 60 min stress by tracking the same cells. The applied stress for 60 min resulted in compression of Blebbistatin-treated cell nuclei [Fig. 3(c) and supplementary material, Fig. S4]. This result suggests that compression of the nucleus increased YAP/TAZ nuclear translocation even after disruption of non-muscle myosin II activity, consistent with a recent report that the resultant nuclear compression facilitates YAP nuclear translocation independent of cell contractility and substrate stiffness.^24^ As expected, pretreatment with Colchicine (10 *μ*M for 30 min), a specific disrupter of microtubules (supplementary material, Fig. S5), did not have any effect on short intermittent stress-induced YAP/TAZ translocation [Figs. 4(a) and 4(b)], possibly because Colchicine treatment did not alter levels of F-actin [Figs. 4(c) and 4(d)]. Together, these results suggest that elevated F-actin after short episodes of intermittent stresses induces YAP translocation because of cytoplasmic mechanomemory.

**FIG. 5.**
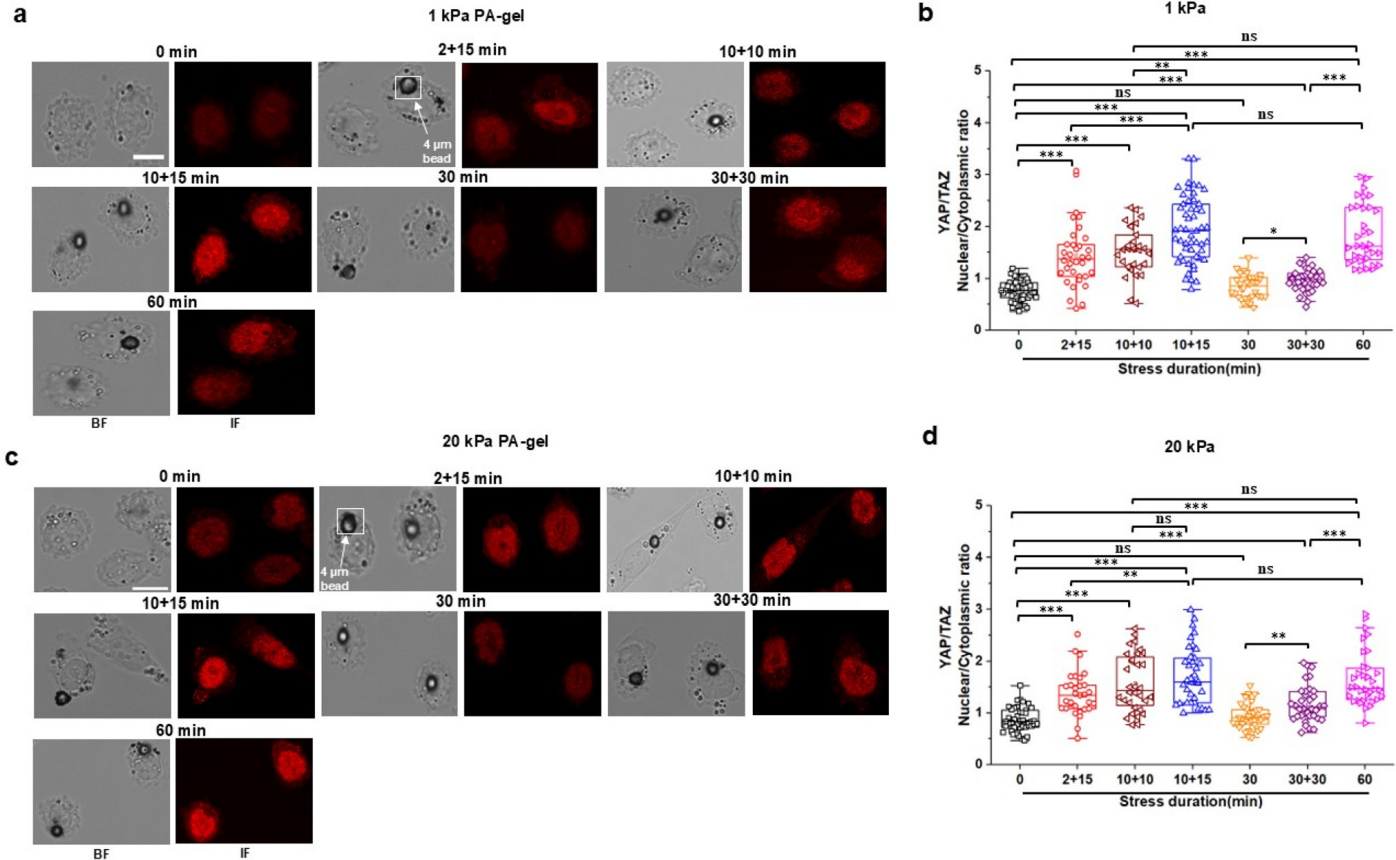
Mechanomemory of YAP/TAZ nuclear translocation on soft and stiff substrates. (a) Representative brightfield (BF) and YAP/TAZ immunofluorescence (IF; red) images (used YAP/TAZ antibody and Anti-Rabbit IgG H&L (Alexa Fluor® 555) antibody) of CHO cells grown on a 1 kPa PA-gel at 0 min (no stress; control), multiple 2 min (2 + 15 min) or 10 min with 10 min (10 + 10 min) or 15 min (10 + 15 min) interval, continuous 30 min or 60, and 30 min interval after 30 min continuous (30 + 30 min) stress of 15 Pa at 0.3 Hz. Circular black dots are RGD-coated ferromagnetic bead. Scale bar, 10 *μ*m. (b) Normalized YAP/TAZ nuclear to cytoplasmic (Nuclear/Cytoplasmic) intensity ratio for 0, 2 + 15, 10 + 10, 10 + 15, 30, 30 + 30, or 60 min applied stress of 15 Pa at 0.3 Hz on 1 kPa PA-gel substrate. Data are shown as boxplot with values of minimum, 5% percentile, 25% percentile, median, 75% percentile, maximum, and mean. n = 49 cells for 0 min, n = 34 cells for 2 + 15 min, n = 29 cells for 10 + 10 min, n = 52 cells for 10 + 15 min, n = 32 cells for 30 min, n = 35 cells for 30 + 30 min, and n = 34 cells for 60 min stress condition on 1 kPa PA-gel substrate from three independent experiments; *P* = 6.40 × 10^−8^ between 0 and 2 + 15 min; *P* = 4.16 × 10^−8^ between 0 and 10 + 10 min; *P* = 1.20 × 10^−8^ between 0 and 10 + 15 min; *P* = 0.0004 between 2 + 15 and 10 + 15 min; *P* = 6.48 × 10^−8^ between 0 and 60 min; *P* = 5.00 × 10^−5^ between 0 and 30 + 30 min; *P* = 0.04 between 30 and 30 + 30 min; *P* = 2.42 × 10^−8^ between 30 + 30 and 60 min; *P* = 0.006 between 10 + 10 min and 10 + 15 min. (c) Representative brightfield (BF) and YAP/TAZ immunofluorescence (IF; red) images (used YAP/TAZ antibody and Anti-Rabbit IgG H&L (Alexa Fluor® 555) antibody) of CHO cells grown on a 20 kPa PA-gel at 0 min (no stress; control), multiple 2 min (2 + 15 min) or 10 min with 10 min (10 + 10 min) or 15 min (10 + 15 min) interval, continuous 30 min or 60, and 30 min interval after 30 min continuous (30 + 30 min) stress of 15 Pa at 0.3 Hz. Circular black dots are RGD-coated ferromagnetic bead. Scale bar, 10 *μ*m. (d) Normalized YAP/TAZ nuclear to cytoplasmic (nuclear/cytoplasmic) intensity ratio for 0, 2 + 15, 10 + 10, 10 + 15, 30, 30 + 30, or 60 min applied stress of 15 Pa at 0.3 Hz on 20 kPa PA-gel substrate. Data are shown as boxplot with values of minimum, 5% percentile, 25% percentile, median, 75% percentile, maximum, and mean. n = 43 cells for 0 min, n = 31 cells for 2 + 15 min, n = 32 cells for 10 + 10 min, n = 37 cells for 10 + 15 min, n = 34 cells for 30 min, n = 34 cells for 30 + 30 min, and n = 34 cells for 60 min stress condition on 20 kPa PA-gel substrate from three independent experiments; *P =* 1.21 × 10^−7^ between 0 and 2 + 15 min; *P* = 4.05 × 10^−8^ between 0 and 10 + 10 min; *P* = 4.24 × 10^−8^ between 0 and 10 + 15 min; *P* = 0.008 between 2 + 15 and 10 + 15 min; *P* = 4.13 × 10^−8^ between 0 and 60 min; *P* = 7.33 × 10^−5^ between 0 and 30 + 30 min; *P* = 0.002 between 30 and 30 + 30 min; *P* = 0.000 03 between 30 + 30 and 60 min.^*^P < 0.05, ^**^P < 0.01, ^***^P < 0.001; ns, not significantly different. All *P* values were quantified using one-way ANOVA with Tukey's test and Mann–Whitney test.

All the above experiments were performed in cells that were plated on Fibronectin-coated rigid glass. To explore the potential effects of substrate stiffness on the cytoplasmic mechanomemory, we plated these cells on substrates of 1 or 20 kPa to simulate soft or stiff substrates. Comparing 1 kPa with 20 kPa, in response to intermittent stresses, as expected, the cells on 20 kPa substrate exhibited more YAP/TAZ translocation than those on 1 kPa substrate prior to the external stress [supplementary material, Fig. S6(a)]. However, the mechanomemory of the cells on the soft (1 kPa) substrate was similar to that on the stiff (20 kPa) substrate [Figs. 5(a)–5(d); supplementary material, Fig. S6(b)], suggesting that under these substrate conditions and for the same stress pattern, the net increase in YAP/TAZ translocation was higher on the 1-kPa substrate than the 20-kPa substrate. In the absence of mechanical stimulation, there was no change in YAP translocation as a function of time for the duration of the experiments in these cells on either the 1-kPa or the 20-kPa substrate (supplementary material, Fig. S7), suggesting that the observed changes in YAP translocation were due to external magnetic bead stress stimulation but not due to temporal changes on those substrates.

## III. DISCUSSION

In the current study, we tested our hypothesis that applications of short intermittent episodes of stress exert long-term effects on mechanotransduction via cytoplasmic mechanomemory. Our results show that short multiple episodes of intermittent 2 or 10 min stresses resulted in similar elevated YAP/TAZ translocation as the 60 min continuous stress application on substrates of stiffness varying from 1 kPa (brain-tissue-like stiffness) to 20 kPa (skeletal muscle-tissue-like stiffness) to ∼2-GPa - rigid glass (calcified-bone-like stiffness). The elevated YAP/TAZ translocation due to intermittent external stresses depends on the external-stress-increased F-actin in the cytoplasm and the cytoskeletal prestress but does not depend on the integrity of the microtubules. The elevated translocation of YAP/TAZ into the nucleus could have implications for the long-term biological functions of the cells.

How does elevated F-actin lead to YAP/TAZ translocation into the nucleus? Elevated F-actin due to external stress likely binds to Lats1/2 (large tumor suppressor kinase 1 and 2). When F-actin is bound to Lats1/2, YAP/TAZ stays unphosphorylated, leading to translocation of the YAP/TAZ to the nucleus. When F-actin is disrupted, Lats1/2 is activated, and the activated Lats1/2 phosphorylates YAP/TAZ.^25^ Phosphorylated YAP/TAZ stays in the cytoplasm and cannot translocate into the nucleus. This hypothesis was supported by the results of elevations of levels of Lats1/2 mRNA (supplementary material, Fig. S8), Lats1 protein, and Lats1 phosphorylated protein (supplementary material, Fig. S9) after treatment with LatA and Blebbistatin to disrupt F-actin and/or actomyosin interaction, but not after treatment with colchicine.

Many types of cells in living tissues in the body experience extracellular matrices of different stiffnesses, ranging from extremely soft in the bone marrow to relatively rigid in the bone.^26^ It is known that malignant soft tumor-repopulating cells in soft matrices proliferate, but they become dormant in stiff matrices.^27^ The results show that intermittent stress causes further YAP translocations. This suggests that these soft cells on soft matrices may be more responsive to intermittent stresses than on stiff matrices by elevating YAP translocation. If this were the case, it would be possible to use intermittent stresses to induce stem-cell like tumor-repopulating cells in soft matrices to undergo YAP-dependent differentiation. This hypothesis needs to be examined experimentally in the future.

### A. Limitations of the current study

In the current study, we only quantified the YAP/TAZ immunofluorescence intensity at one focal plane (at the maximum nuclear projection area). Because the cell is a 3D structure, the total amount of the changes in YAP/TAZ translocation could be higher than changes in YAP/TAZ from the two-dimensional (2D) measurements. However, the CHO cells are very spread in the present study, suggesting the z-dimension of the cells is much smaller than their x-y dimensions. Similarly, the F-actin intensity is only quantified at the maximum cell spreading area near the basal surface. This 2D F-actin intensity measurement might underestimate the total changes in F-actin in response to intermittent external stresses. In addition, we applied the stresses to the cells only at one amplitude and one frequency (15 Pa at 0.3 Hz, which is within the range of physiological magnitudes and frequencies). It is possible that cellular responses to the intermittent stresses at different stress amplitudes, frequencies, durations, and stress-free pauses might be different. Therefore, these variables should be systematically examined in detail in future studies. Furthermore, focal adhesion proteins such as talin are known to exhibit mechanomemory in response to externally applied stresses.^28^ It is not clear at this time to what degree the mechanomemory at the focal adhesions contributes to the integrated overall cellular responses to the external stress long after cessation of the stress, but the YAP/TAZ translocation data after the 60 min continuous stress application in the presence of the Blebbistatin in our current study suggest that focal adhesion mechanomemory might play a role there. Finally, because the intermittent stresses via the integrins on the cell surface also cause significant nuclear deformation and chromatin strains, leading to nuclear mechanomemory,^18,19^ it will be interesting to investigate how cells integrate the YAP/TAZ translocation mediated nuclear responses with the chromatin-stretching dependent nuclear mechanomemory.

Is the magnitude of 15-Pa stress from the MTC relevant to the stresses *in vivo*? Shear stresses in blood vessels are about 1 Pa. Attachments of developing skeletal muscle cells *in vivo* in *Drosophila* generate ∼500 Pa stresses.^29^ Zebrafish embryos *in vivo* generate oscillatory stresses of several hundred Pa.^30^ The applied oscillatory stresses via MTC in this study are 10-fold higher than the blood shear stress but are an order of magnitude lower than the tractional stresses in developing embryos and hence are within the range of physiologically relevant stresses.

### B. Significance

Here, we have presented experimental evidence that cumulative short episodes of intermittent stresses can cause YAP translocation, which might result in long-term effects on cell functions and behaviors in the epithelial cell line of CHO cells. It would be tempting to speculate whether this finding could be extended to *in vivo* exercise, where mechanical stresses are exerted on tissue cells through skeletal muscle contractions. It is possible that multiple short intermittent (say, 2 or 10 min) exercises may achieve the same effect as the continuous long (60 min) durations of exercises, providing a cellular and subcellular mechanism for the published report of long-term effects of high-intensity interval training in optimizing physiological performance at the level of the whole human body.^31^ In early published studies, workout durations are varied from 5 s to 30 min, and rest durations are varied from 0 s to ∼60 min, covering a wide range of stress periods and stress-free periods, with no clear evidence and no mechanisms of whether some particular regimens are more effective or less effective. Our experimental results suggest that at the cellular level, 2- or 10 min stress and 10–30 min stress-free periods might be optimal for producing long-term biological effects on cellular responses through the mechanisms of cytoplasmic (and nuclear) memory, producing long-term impacts on cellular functions such as gene expression and protein synthesis. Our results at the single cell level are also consistent with the finding that long (>10.6 h) durations of sedentary behaviors are associated with future adverse cardiovascular outcomes,^32^ possibly because of diminishing mechanomemory effects on the physiological functions of tissues and organs. A recent report finds that multiple pulses of soluble molecules induce memory in non-neural cells.^33^ We anticipate that multiple episodes of intermittent force-induced mechanomemory, together with memory that is induced by multiple pulses of soluble molecules, may dramatically influence behaviors and fates of living cells. Our current findings on cellular mechanomemory are significant in that they may have implications in mechanomedicine and mechanohealth.

## IV. METHODS

### A. Cell culture

In this study, dihydrofolate reductase (*DHFR*) BAC (bacterial artificial chromosome) containing Chinese Hamster Ovary (CHO) DG44 cells were used. These *DHFR* BAC inserted CHO DG44 cells were cultured with 10% dialyzed FBS (Fetal Bovine Serum) in Ham's F12 medium without hypoxanthine and thymidine as described previously.^18,19,34,35^ These cells stably expressed EGFP-Lac Repressor (GFP-LacI) that binds with *DHFR* gene tagged with LacO (256-mer Lac Operator) to visualize the transgene in live cell chromatin.^18,19,34^ These CHO DG44 cells were grown in a humidified incubator at 37 °C and 5% CO_2_ and passaged every 3 days using TrypLE Express (Thermo Fisher Scientific). For all biological experiments, CHO DG44 cells were randomly seeded onto different culture dishes. During the cell culture and experiments, mycoplasma contamination was monitored using DMIRE2 Leica inverted microscope and staining with 4′,6-diamidino-2-phenylindole (DAPI). There was no mycoplasma contamination during cell culture and experiments for CHO DG44 cells.

### B. Ferromagnetic bead coating

In this study, RGD (Arginine-Glycine-Aspartic acid)-coated 4-*μ*m ferromagnetic beads were used to apply different short episodes of intermittent and continuous stresses. These ferromagnetic beads were obtained from Dr. Jeffrey Fredberg's laboratory (Boston, MA), and RGD-coating was conducted using the method described previously.^36^ In brief, after washing with PBS (phosphate-buffered saline), 4-*μ*m ferromagnetic beads were centrifuged and resuspended in 1 ml carbonate buffer (pH 9.4). RGD-peptides were added to ferromagnetic beads solution with a final concentration of 50 *μ*g/ml and mixed with the beads overnight on a rotator at 4 °C. These RGD-coated ferromagnetic beads were bound to integrin cell surface receptors to apply different short episodes of intermittent and continuous stresses.

### C. Magnetic twisting cytometry (MTC)

Three-dimensional (3D) magnetic twisting cytometry (MTC) can generate a strong magnetizing field (∼1000 Gauss) to magnetize ferromagnetic material in one axis direction (X, Y, or Z) and apply a weak sinusoidal (oscillatory) twisting field (0–50 Gauss) orthogonally to exert in- and out-of-plane rotation of these two axes.^18,19,34,37^ In this study, one-dimensional (1D) MTC with two pairs of coils aligned along the Y- and Z-axis directions was used. This 1D MTC was used to magnetize (∼1000 Gauss, ∼10 ms) RGD-coated 4-*μ*m ferromagnetic beads in the Y-direction and apply a sinusoidal twisting field of 50 Gauss at 0.3 Hz in the Z-axis direction and exert a magnetic field directly to integrin cell surface receptors.

First, about 100, 000 CHO DG44 cells were seeded on a Fibronectin (5 *μ*g/ml; Thermo Fisher Scientific)-coated 35-mm dish (GBD00001-200, Cell E&G, San Diego, CA). After overnight (12 h) incubation, ∼2 × 10^5^ RGD-coated ferromagnetic beads (30 *μ*l of RGD-coated beads; 1 mg ferromagnetic beads per 1 ml of cell culture medium) were added and incubated for ∼30 min in a humidified incubator at 37 °C and 5% CO_2_. After incubation, cells were rinsed twice with fresh cell culture medium to remove unbound RGD-coated 4-*μ*m beads. Finally, a 2 ml cell culture medium with HEPES buffer (10 mM; Thermo Fisher Scientific) was added before applying different short episodes of intermittent and continuous stresses. Cells with a single RGD-coated 4-*μ*m bead were taken for all experimental analyses to avoid complications of stresses from multiple beads on a single cell. Cells with no beads were considered as a control for all experimental analyses.

For short episodes of intermittent and continuous stress, CHO DG44 cell dishes were placed on a 1D MTC chamber with two pairs of coils. For 2 + 15 min intermittent stress, after magnetizing along the Y-axis direction with a strong magnetizing field of about 1000 Gauss for 10 ms and applying a twisting field (50 Gauss at 0.3 Hz) along the Z-direction for four episodes of intermittent 2 min stresses with four intervals of 15 min of load-free period for a total duration of 68 min before starting the experiment. Similarly, for 10 + 15 min intermittent stress, a sinusoidal twisting field (50 Gauss at 0.3 Hz) was applied for three episodes of intermittent 10 min stresses with two intervals of 15 min of load-free period for a total duration of 60 min. For 30 + 30 min intermittent stress, one twisting field (50 Gauss at 0.3 Hz) of 30 min stress was applied with a 30 min load-free period for a total time duration of 60 min before starting the experiment.

On the other hand, for continuous stress, one 30 min or one 60 min continuous twisting field (50 Gauss at 0.3 Hz) was applied, and the experiment started immediately without any load-free period. These conditions were considered as controls. For both intermittent and continuous twisting field (50 Gauss at 0.3 Hz) patterns, the magnetic moment constant for a 4-*μ*m bead is 0.3 Pa per Gauss, which corresponds to 15 Pa stress.

### D. Immunofluorescence and western blot

After adding RGD-coated 4-*μ*m ferromagnetic beads and applying all six different patterns of stress durations, CHO DG44 cells were fixed using 4% paraformaldehyde (Santa Cruz) at room temperature for 10 min. After that, cells were rinsed three times with phosphate-buffered saline (1X PBS) and permeabilized using Triton X-100 (Sigma Aldrich; 0.3% Triton X-100 in PBS) at room temperature for 20 min. These permeabilized cells were treated with normal donkey serum (5% (v/v) in PBS; Jackson ImmunoResearch Laboratories Inc.) for 4 h for blocking at room temperature. After that, cells were incubated overnight (12 h) with primary antibody (rabbit monoclonal Anti-YAP/TAZ (Catalog#8418; Cell Signaling Technology), diluted 1:100 (v/v); Phospho-Myosin Light Chain 2 (Ser19) (Catalog#3671; Cell Signaling Technology), diluted 1:1000 (v/v) in 1% BSA) at 4 °C. After overnight (12 h) incubation, cells were rinsed three times with 1X PBS each time for 5 min. Incubation with secondary antibody (donkey Anti-Rabbit IgG H&L, Alexa Fluor 555, Catalog#ab150062, Abcam; 1:200 (v/v) dilution in PBS) was done for about 2 h at 4 °C. Finally, cells were rinsed three times with PBS before proceeding to imaging experiments. The data analysis for immunofluorescence imaging experiments was performed using ImageJ. After subtracting average background fluorescence, the integrated density of fluorescent nuclear and cytoplasmic areas was quantified to calculate YAP/TAZ translocation.

For Western blot, YAP/TAZ Rabbit monoclonal antibody (D24E4; 1:1000 dilution, v/v, Cell Signaling, #8418), Rabbit monoclonal anti-LATS1 (Cell signaling, #3477, 1:2000 dilution), Rabbit monoclonal anti-pLATS1(T1079) (D57D3) (Cell signaling, #8654, 1:1000), anti-CTGF antibody [1:1000 dilution, abcam (EPR20728)], GAPDH rabbit polyclonal antibody (1:1000 dilution, v/v, Sigma, G9545), and goat Anti-Rabbit IgG (HRP-linked, 1: 1000 dilution, v/v, Cell Signaling, #7074) were used. For nuclear and cytoplasmic YAP/TAZ relative expression quantification, we used NE-PER™ Nuclear and Cytoplasmic Extraction Reagents (Thermo Fisher Scientific) for subcellular fractionation. After extracting protein samples and performing the experiments, we conducted imaging processes with BIO-RAD Chemidoc^TM^ MP, and relative expressions were quantified using Image Lab software.

### E. Live cell F-actin, nuclear staining, and imaging

For live cell F-actin imaging, after adding RGD-coated 4-*μ*m ferromagnetic beads, CHO DG44 cells were stained with SiR-actin KIT (Catalog# CY-SC001; Cytoskeleton, Inc.) by following the previously mentioned method.^38^ Briefly, cells were incubated with 0.1% (v/v) SiR-Actin KIT diluted in cell culture medium for 4 h in a humidified incubator at 37 °C and 5% CO_2_. After that, cell dishes were placed in the MTC chamber, and all six different patterns of stress durations were applied and proceeded for imaging.

For F-actin imaging experiments, cell dishes were placed on a stage incubator (Okolab on stage incubator; Leica Microsystems), and fluorescence imaging was conducted in an X-Y plane. Because CHO DG44 cells were very spread, cell dimensions along the Z-direction were smaller than those along the X-Y plane.

For nuclear staining, Hoechst Stain (Catalog#H1399; Thermo Fisher Scientific) was used for cells treated with 20 *μ*M Blebbistatin. Cells were seeded and tracked on a 35-mm glass-bottomed gridded dish (81148, Ibidi USA Incorporated, Madison, WI) before, after 20 *μ*M Blebbistatin (for 30 min) treatment, and after 1 h stress.

F-actin fluorescence intensity and nuclear area were quantified using ImageJ. In brief, after subtracting average background fluorescence, the integrated density of fluorescent cell areas was quantified to calculate the elevation of F-actin intensity with different patterns of stress durations, and the nuclear area was quantified before and after Blebbistatin treatment.

### F. F-actin, microtubule, and actomyosin contractility disruption

In this study, YAP/TAZ translocation was quantified after disrupting F-actin, microtubule, or actomyosin contractility. Disruption of F-actin, microtubules, and actomyosin contractility was conducted following the method described previously.^38^

For F-actin disruption, after adding RGD-coated 4-*μ*m ferromagnetic beads, cells were incubated with 1 μM Latrunculin A (Catalog#100-0563; STEMCELL Technologies) for 30 min in a humidified incubator at 37 °C and 5% CO_2_. To quantify F-actin disruption after 30 min treatment with Latrunculin A, cell dishes were placed on a stage incubator (Okolab on stage incubator; Leica Microsystems) before proceeded for fluorescence imaging experiments. To track individual cells before and after Latrunculin A treatment, CHO DG44 cells were grown on a Fibronectin (5 μg/ml)-coated 35-mm gridded glass-bottomed dish (Catalog#81148, Ibidi USA Incorporated, Madison, WI). To quantify YAP/TAZ translocation, cell dishes were placed in the MTC chamber, and six different patterns of stress durations were applied after 30 min treatment with Latrunculin A. Then, samples were prepared for the immunofluorescence imaging experiment.

For microtubule disruption, cells were incubated with 10 μM colchicine (Catalog# HY-16569; MedChem Express Co. Ltd) for 30 min in a humidified incubator at 37 °C and 5% CO_2_. To quantify microtubule disruption after 30 min treatment with colchicine, cell dishes were placed on a stage incubator (Leica Microsystems) and proceeded for fluorescence imaging experiments. To quantify YAP/TAZ translocation after microtubule disruption, cell dishes were placed in the MTC chamber and samples prepared for the immunofluorescence imaging experiment after stress application.

Cells were treated with 20 μM Blebbistatin (Catalog# B1387; ApexBio Tech LLC) to inhibit actomyosin contractility (Myosin inhibitor of non-muscle myosin II ATPase) after adding RGD-coated 4-*μ*m ferromagnetic beads. For YAP translocation experiments, after inhibiting the myosin inhibitor of non-muscle myosin II ATPase, cell dishes were placed in the MTC chamber, and six different patterns of stress durations were applied after 30 min treatment with Blebbistatin. Then, samples were prepared for the immunofluorescence imaging experiment.

### G. Polyacrylamide (PA) gel preparation

To find the effect of elastic substrate stiffness with different patterns of stress durations on YAP/TAZ translocation, CHO DG44 cells were seeded on Fibronectin (30 mg per mL)-coated Polyacrylamide (PA) gels of 1 and 20 kPa. This 1 and 20 kPa PA-gel substrates were prepared following the published method.^39^ Briefly, by varying the mixture volume and concentrations of 2% Bis-acrylamide (Catalog #161-0142; Biorad) and 40% acrylamide (Catalog #1610140; Biorad) with water, 1 and 20 kPa PA-gel substrates were prepared. More specifically, to prepare a 1 kPa PA-gel solution, 1.25 ml of 40% acrylamide and 0.15 ml of 2% Bis-acrylamide solutions were mixed with water to make a total volume of 10 ml. Similarly, a 20 kPa gel solution was prepared by mixing 2.5 ml of 40% acrylamide and 0.934 ml of 2% Bis-acrylamide with water to make a total volume of 10 ml. Tetramethylethylenediamine (TEMED) and 10% (w/v) ammonium persulfate (APS) were used for the polymerization of these 1 and 20 kPa PA-gel substrates.

To quantify the efficacy of Blebbistatin treatment, we seeded the cells on 1 kPa PA-gel substrate, and we quantified cell tractions before and 30 min after being treated with 20 *μ*M Blebbistatin at 0 min stress.

### H. Microscopy and live cell imaging

For all imaging and MTC experiments, a 63X oil objective of a numerical aperture of 1.32 was used in a DMIRE2 Leica inverted epifluorescence microscope, and a 63X oil objective of a numerical aperture of 1.40 was used in a Leica DMi8 CS Premium (STELLARIS 5) confocal microscope. For the PA-gel substrate imaging experiment, both 40X air (NA = 0.95) and 63X oil (NA = 1.40) objectives were used. Leica DMIRE2 and DMi8 (STELLARIS 5) confocal microscopes were used for fluorescence imaging with YAP/TAZ excited at 555 nm and SiR-actin for live cell F-actin at 633 nm. For all fluorescence imaging experiments, HyD S detectors were used in the Leica DMi8 (STELLARIS) confocal microscope.

### I. Quantitative polymerase chain reaction (qPCR)

We used SsoAdvanced Universal SYBR Green Supermix (Bio-Rad, #1725271) by using CFX Connect Real-Time PCR Detection System (Bio-Rad) for Quantitative polymerase chain reaction (qPCR) measurements. All primers were designed and custom-made from Sigma Aldrich. The primer sequences used for this work are given as follows:

CHO *Ctgf* primer set:
Forward primer: TGTGTGATGAGCCCAAGGAT
Reverse primer: TGCTTCTCCAGTCTGCAGAA
Lats1 primer set:
Forward primer: TGGGACAACCTCCTTTCTTGGC
Reverse primer: TGAGGTCAGAGGCTTCAGGACT
Lats2 primer set:
Forward primer: TCTTCCAACAGCAAGCACAC
Reverse primer: AAGCTCCAGTCTGATCCACC.

To analyze qPCR data, we used ΔΔCt method for the measurements of normalized expression.

### J. Software and data analysis

Data analysis for all imaging experiments was done using ImageJ and MATLAB.

### K. Statistical analysis

All statistical analyses were done using Mann–Whitney and one-way ANOVA with the Tukey test.

## SUPPLEMENTARY MATERIAL

See the supplementary material (Figs. S1–S9) for additional information.

## Data Availability

The data that support the findings of this study are available from the corresponding author upon reasonable request.
